# Observations on Copy Number Variations in a Kidney-yang Deficiency Syndrome Family

**DOI:** 10.1093/ecam/neq069

**Published:** 2011-06-16

**Authors:** Wei Wei Liu, Yong Xiang Gao, Li Ping Zhou, Azure Duan, Ling Ling Tan, Wan Zhen Li, Min Yan, Hong Ya Yang, Shi Lin Yan, Mi Qu Wang, Wei Jun Ding

**Affiliations:** Department of Fundamental Medicine, Chengdu University of Traditional Chinese Medicine, Chengdu 610075, China

## Abstract

We have performed an analysis of a family with kidney-yang deficiency syndrome (KDS) in order to determine the structural genomic variations through a novel approach designated as “copy number variants” (CNVs). Twelve KDS subjects and three healthy spouses from this family were included in this study. Genomic DNA samples were genotyped utilizing an Affymetrix 100 K single nucleotide polymorphism array, and CNVs were identified by Copy Number Algorithm (CNAT4.0, Affymetrix). Our results demonstrate that 447 deleted and 476 duplicated CNVs are shared among KDS subjects within the family. The homologus ratio of deleted CNVs was as high as 99.78%. One-copy-duplicated CNVs display mid-range homology. For two copies of duplicated CNVs (CNV_4_), a markedly heterologous ratio was observed. Therefore, with the important exception of CNV_4_, our data shows that CNVs shared among KDS subjects display typical Mendelian inheritance. A total of 113 genes with established functions were identified from the CNV flanks; significantly enriched genes surrounding CNVs may contribute to certain adaptive benefit. These genes could be classified into categories including: binding and transporter, cell cycle, signal transduction, biogenesis, nerve development, metabolism regulation and immune response. They can also be included into three pathways, that is, signal transduction, metabolic processes and immunological networks. Particularly, the results reported here are consistent with the extensive impairments observed in KDS patients, involving the mass-energy-information-carrying network. In conclusion, this article provides the first set of CNVs from KDS patients that will facilitate our further understanding of the genetic basis of KDS and will allow novel strategies for a rational therapy of this disease.

## 1. Introduction

In order to observe the architectural complexity and structural variations of the human genome, single nucleotide polymorphism (SNP) platforms have recently become a promising approach for both SNP and non-SNP variability [1–3]. Non-SNP variations usually include copy number variants (CNVs), loss of heterozygosity, inversions, insertions, deletions and other complex rearrangements, most of which can not be detected by DNA sequencing [2]. A thorough understanding of the roles of structural genomic variants, such as CNVs, is an important prerequisite to unravel the intricate genetic basis of complex diseases. For example, there is increasing evidence of how CNVs can influence susceptibility to HIV infection [4], modulate drug responses [5] and contribute to genomic microdeletion and duplication syndromes [6]. Beside SNPs, CNVs are now being recognized as another important indicator for inter-individual differences [7]. CNVs can cause Mendelian or sporadic traits, or be associated with complex diseases, by mechanisms such as gene dosage, gene disruption, gene fusion and position effects [8]. Orozco and colleagues [9] observed that gene expression was altered in genes flanking CNVs, suggesting that CNVs may contain regulatory elements for these nearby genes. Therefore, the contribution of CNVs to genetic variation and the consequently impact of diverse phenotypes resulting from the affects of the CNVs to complex diseases may be greater than previously estimated [2, 7–10].

Syndromes defined by traditional Chinese medicine (TCM), such as kidney-yang deficiency syndrome (KDS), share some genetic features with complex diseases and/or disease susceptibility. TCM, by its sheer nature, is based on the integrated insight of diverse syndromes, that is, the simultaneous manifestation of pathological processes on a macroscopic level and thus provides the holistic approach which might counteract or balance the conventional bias of conventional medicine [11–13]. While there has been significant research on the physiology of KDS, few studies have been conducted on its genetic background, largely due to the lagged development of appropriate methodology for the extreme complexity of the genetic basis of TCM syndromes [1, 14, 15]. In contrast, there have been several studies that strongly suggest the genetic basis of KDS. First, the kidneys in TCM are viewed as the root of life activities and represent the origin of our congenital or inherited foundation [1]. They regulate reproductive, urinary, endocrine, skeletal, blood and central nervous functions while also storing primordial yin and primordial yang (known as the inherited kidney yin and yang). Therefore, a congenital deficiency is one of the critical causes of KDS [1, 14–16]. Secondly, relevant studies have shed light on the complex patterns of the transcriptomes of KDS patients [14–16]. Finally, epidemiological and clinical observations have indicated that the development of KDS can be influenced by both genetic and environmental factors [16].

In the present work, we hypothesize that CNVs represent part of the complex genetic basis of KDS. Based on current advances in genome-wide analysis methods (e.g., SNP arrays and their successful application in the genetic exploration of complex diseases), we propose that KDS involves structural variations of genome such as CNVs [1]. In order to prove our hypothesis, we recruited a typical KDS family and employed SNP arrays to probe their DNA structural heterogeneity. Our findings indicate that this approach may facilitate a further understanding of the genetic basis of KDS and will allow novel strategies for a rational therapy of this disease [17–20].

## 2. Methods

### 2.1. Subjects

This work has been approved by the Ethics Board, Chengdu University of TCM, and all participants provided written informed consent to participate in experiments. A middle-scale epidemiological investigation was conducted for the collection of KDS subjects in Chengdu, China, based on a 40-items scoring table for KDS diagnosis [1, 14, 16]. The diagnostic system had been described previously [16]. Subjects were identified as healthy individuals (total score: ≤5 points) or KDS patients (total score: ≥12 points). In order to upgrade the validity of TCM diagnosis and ascertain the KDS subjects, every participant was diagnosed by five independent TCM physicians with single-blind method, and this procedure was repeated for three consecutive years (from 2003 through 2005), all on the first Saturday of December [14].

**Figure 1:**
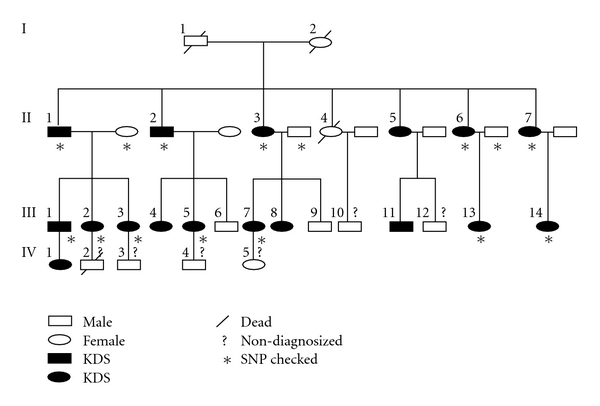
Pedigree tree of the collected KDS family.

This article focuses on a typical KDS family, in which 17 KDS subjects were distributed across four generations. Most members of this family live in surrounding areas of Chengdu, China. Twelve available KDS patients were recruited for the study. Three healthy spouses in the pedigree were also recruited as a non-KDS control group (Figure 1). The living environment, life styles and ages of these non-KDS spouses were best matched with that of the KDS subjects [14].

### 2.2. Isolation and Purification of Genomic DNA Samples

Samples of genomic DNA from all participants were isolated and purified under a standard protocol [1]. Briefly stated, 3 ml of blood was collected from each participant. Following this, the genome DNA was extracted by conventional phenol/chloroform method, purified by 24 : 1 chloroform : isoamyl alcohol and 95% ethanol, re-suspended in TE buffer, and stored at −80°C prior to its usage.

### 2.3. Genotyping, Data Formatting and Quality Control

Genomic DNA samples were genotyped in a commercial laboratory (National Engineering Center for Biochip at Shanghai, China), utilizing the Affymetrix 100 K array ( http://www.affymetrix.com/) according to the manufacturer's instructions. Briefly, SNP genotyping was performed with the HindIII array of the Affymetrix GeneChip Mapping 100 K set. The arrays were hybridized, washed and scanned as per the manufacturer's instructions. Genotype data of the individual family members was generated using GeneChip DNA Analysis Software (GDAS, Affymetrix). The pedigree information, allele frequencies and map position of the SNPs were combined with the genotype data generated by GDAS [1].

### 2.4. Analysis of CNVs

We employed a software, Copy Number Algorithm (CNAT4.0, Affymetrix), for obtaining the shared CNVs among KDS subjects from the KDS family. This software is especially useful for the SNP analysis of complex diseases [14, 17–20]. The data-mining procedure was performed with the following steps:

1. Probe-level and SNP-level filtering.
2. Probe-level normalization of signal intensity.
3. Allele-specific summarization for each SNP.
4. Global reference generation for unpaired experiments.
5. Raw copy number estimation.
6. Linear regression on the raw copy number estimate to correct for artifacts introduced by the polymerase chain reaction (PCR) fragmentation process.
7. PCR normalized copy number data—Gaussian smoothed for enhancement of the signal-to-noise ratio (SNR). All data based on the PCR results with a primer (5′ATTATGAGCACGACAGACGCCTGATCT 3′) were automatically normalized by method of Gaussian smoothed Log2 ratio value for the allele with the higher signal intensity.
8. Hidden Markov model (HMM)-based segmentation to obtain the different CNV state partitions and median log_2_ ratio value of all the contiguous SNPs in the given HMM copy number state segment of the allele with the higher signal intensity.

The parameters used for CNV designation required that four SNPs on three restriction fragments gave rise to a signal-intensity ratio >1.12 for insertions or >0.89 for deletions. CNVs were considered significant for *P*-values <.01 using 5000 permutations of the data. For data integration, only CNVs identified in at least 10% of the comparisons to the diploid samples were retained [20].

### 2.5. Mining the Genes Located in the Flanks of CNVs

Genes located within 100 bp of the CNVs' flanks [8, 20] were checked via an authoritative Web tool, GeneView ( http://www.ncbi.nlm.nih.gov/SNP/). Their biological functions and related pathways were then annoted by FatiGO ( http://fatigo.bioinfo.ochoa.fib/es) and KEGG ( http://www.kegg.jp/kegg/pathway.html), respectively.

## 3. Results

### 3.1. A Representative KDS Family Collected by TCM Diagnosis

We collected a typical KDS pedigree, including 17 KDS subjects who live around Chengdu, China. Twelve available KDS patients and three healthy spouses (as non-KDS control) of the particular pedigree were collected for the identification of CNVs. Our selection criteria indicated that almost all of the scores marked by five physicians tallied with each other, and scores marked from 2003 through 2005 for every participant were reproducible [1, 14], indicating the reliability of the classification of the recruited subjects and the validity of the employed TCM criteria. Hence, these samples were utilized for identifying CNVs related to KDS (Figure 1).

### 3.2. Identification of Shared CNVs among KDS Subjects

**Figure 2:**
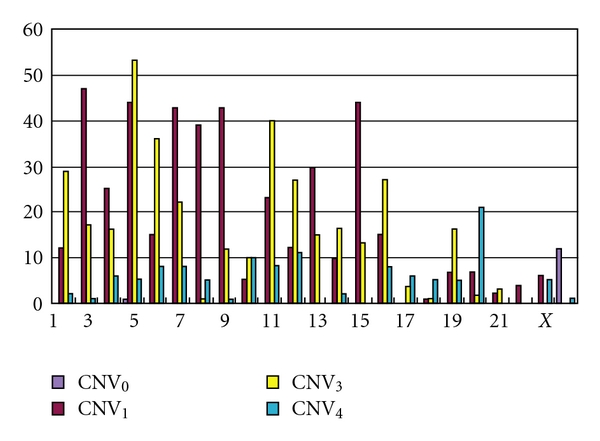
The chromosome distribution of shared CNVs among KDS patients.

Results of SNP arrays revealed thousands of CNVs from KDS patients. Among them, 447 of deleted and 476 of duplicated CNVs are shared among all KDS subjects within the family analyzed in this study. These CNVs are not equally distributed among chromosomes (Figure 2). CNVs that totally deleted (CNV_0_) are mostly located on chromosome X. One-copy-deleted CNVs (CNV_1_) are frequently distributed on chromosomes 2, 4, 6–8 and 14, and a few were observed on chromosomes 9, 16–22 and X. CNVs such as one-copy-duplicated CNV_3_ are frequently located on chromosomes 4, 5 and 10. Tetraplont CNVs (CNV_4_) are principally observed on chromosomes 9, 11 and 19.

These CNVs were then analyzed for Mendelian inheritance. Our results indicated that only one of the 447 deleted CNVs was not consistent with those of other family members (Table 1). The homologous ratio of deleted CNVs was as high as 99.78%; thus indicating that the deleted CNVs are clearly inherited in this KDS family. In contrast, the duplicated CNVs (i.e., gain of one or two copies) display a much lower homologous ratio (62.61%).

### 3.3. Functional Annotation of Genes Located within the Flanks of Shared CNVs

A total of 113 genes with established functions were identified from the flanks of shared CNVs. Of these, 41 genes were derived from the deleted CNVs, and can be subtyped into five functional classes: binding and transporter, cell cycle, cell adhesion, signal transduction and immune-response genes. The 72 genes derived from duplicated CNVs can be classified into five functional groups: binding and transporter, nervous system development, metabolism regulation, biogenesis and immune-response genes (Table 2).

Pathways related to the genes located in the CNV flanks can largely be classified into three groups: signal transduction pathways, immune-response pathways and metabolic processes. The mitogen-activated protein kinase (MAPK) pathway, transmembrane receptor protein tyrosine kinase signaling pathways and neuropeptide signaling pathways all belong to signal-transduction pathways and these pathways are concordant with the relevant results of the transcriptome analysis of KDS [14]. Pathways such as Fc epsilon RI signaling, cytokine-cytokine receptor interactions, T cell receptor signaling pathways, and antigen processing and presentation are major immune-response pathways. Other pathways are largely involved in metabolic processes and energy metabolism (Table 3).

## 4. Discussion

Our results are consistent with previous studies indicating that CNVs are highly inheritable [8, 17–20]. We used an established platform to identify CNVs which has been extensively utilized for identification of both linkage-disequilibrium SNPs and CNVs [17–20]. Totally there are 923 CNVs shared among KDS subjects from a single family. The high homologous ratio of deleted CNVs suggests a typical Mendelian inheritance within the KDS family. Similarly, the one-copy-duplicated CNVs show moderate Mendelian inheritance. Thus, we conclude that the majority of CNVs in KDS offspring are inherited, confirming previous reports that CNVs are highly inheritable [21–24]. Thus, our work displays a clear Mendelian pattern of inheritance for KDS, a TCM syndrome that shares many features with complex diseases [1, 14, 16]. This result and relevant studies by other groups indicate that many larger scale variations, such as CNVs, may represent a major genetic component of phenotypic diversity [25–30]. Therefore, CNVs should be considered another kind of relevant markers for use in the genetic exploration of complex diseases as well as TCM syndromes.

In contrast to the strong correlation of specific CNVs with inheritance, a significant difference was found with two-copy-duplicated CNVs (CNV_4_) as these CNVs show Mendelian inconsistency rather than inheritance. Among the 113 CNV_4_ derived from this KDS family, 93 were heterologous, implying that 82.30% of these CNV_4_ were new mutations. However, the exact mechanisms of individual mutations and the consequences of these mutations on the disease process are not understood. Theoretically, CNV_4_ should be less stable than those of other CNVs [29–31]. Consequently, from an evolutionary standpoint, the low degree of CNV_4_ inheritance might reinforce the concept of chromosome stability in this disease phenotype. Indeed, the functional impact of most CNVs remains poorly characterized [32, 33] and our current knowledge regarding CNVs and their heritability is still rudimentary, due to their location in regions of complex genomic structure and to the technical limitations of association studies [34, 35]. Taken together, with the important exception of CNV_4_, our data suggest that CNVs shared among KDS patients display typical Mendelian inheritance.

CNVs can intensively impact the activity of nearby genes. Orozco et al. [9] observed that gene expression was altered in genes flanking CNVs; suggesting that CNVs may contain regulatory elements for these genes, and may play a role in the mechanisms underlying specific metabolic traits in mice. Our results identified a significant overabundance of genes in the flanking region of CNVs which are shared among KDS subjects. A total of 113 genes with established functions were identified within 100 bp of the CNV flanks, representing 12.24% of the CNVs (113/923). Considering the fact that <1% of the sequence of human genome is composed of genes, our data show the first set of markedly enriched protein-coding genes in the CNV flanks [23]. The excess overabundance of genes surrounding CNVs may be due to an adaptive benefit of increased gene dosage, particularly in those genes involved in fighting infection and sensing the environment [21–24].

Bioinformatic analysis show that genes located in the flanks of CNVs of KDS patients can be classified into a small number of classes. Genes related to deleted CNVs largely belong to binding and transporters, the cell cycle, signal transduction and the immune response, whereas genes associated with duplicated CNVs, can be categorized into binding and transporters, biogenesis, nervous system development, metabolism regulation and the immune response. Therefore, these findings indicate that many vital physiological functions, such as metabolism, biogenesis, development, signal transduction and the immune response are ubiquitously attenuated or disrupted in KDS patients.

In the TCM overview, the kidney is in charge of vital body functions and its role is broader than just the anatomical kidney [16]. It performs reproductive, urinary, endocrine, skeletal, blood and central nervous functions while also storing primordial yin and yang. Hence, the TCM Kidney is the most important system of the body. It is the root of life activities, warms and promotes the functions of other organs and is the origin of our congenital (inherited) foundation [1, 11, 16]. Since the TCM kidney affects so many vital functions, KDS patients present with a wide range of symptoms, including: sore and/or pain in the lower back, cold and weak limbs, water retention in the legs, copious and pale urine, aversion to cold, poor appetite, spiritual fatigue, depression, sexual dysfunction, premature ejaculation, adrenal fatigue, hypothyroidism and a weak pulse. In short, Kidney yang is the pilot light for our energy system; and the diming of this light represents KDS and consequently results in the insufficient function of the reproductive, urinary, endocrine, skeletal and nervous systems.

Our results indicate that CNVs derived from KDS subjects are enriched in genes that are consistent with KDS symptoms. Based on the results presented here, we offer the following conclusions. First, KDS patients show an extensively attenuated ability to resist infection and to adapt to their environment, in accordance with the TCM theory that “Kidney yang prevents evils or pathogens from invading the body” [14, 16]. More importantly, firstly, Kidney yang works closely with the neural-endocrine-immune network thus connecting the immune response to the environment. Second, KDS patients demonstrate the potential for altered gene expression in those genes involved in the cell cycle, biogenesis and nervous system development, aligning with the TCM concept that “the kidney is the regulator of growth and development”. Third, genes involved in metabolism regulation are overrepresented in the CNV flanks of KDS subjects, a finding in agreement with another TCM principle that “Kidney yang motivates the power of human vitality, and controls the storage of vital essence”. In view of these identified CNVs, many vital body activities of KDS patients might be attenuated or disrupted. In brief, the results of our CNV analysis provide a basis for the systemic disorders of KDS patients.

It is established that pedigree is the most classical approach applied in human genetics studies in conventional medicine. However, few studies have been performed on the exploration of genetic traits of TCM syndromes by the pedigree approach. In this study, we recruited a typical KDS family of Han Chinese, compared the different SNPs between 12 KDS and three Non-KDS spouses within the family and identified 923 CNVs shared among the KDS subjects. This design of CNV research has also been successfully employed in previous publications from other laboratories. For instance, Yang et al. [17] genotyped 46 individuals in a three-generation pedigree with 19 affected and 27 unaffected subjects, and identified 50 CNV regions. Therefore, we conclude that the pedigree approach is reasonable and suitable for genetic studies of TCM syndromes.

The main limitation of this work is that only one KDS family was screened for the presence of CNVs. Theoretically, the ultimate proof of the involvement of CNVs in TCM syndromes will require large-scale studies of multiple families. Therefore, the limitation of this report can be overcome by introducing a similar design of other KDS pedigrees and KDS patients collected via a randomized method of identification.

**Figure 3:**
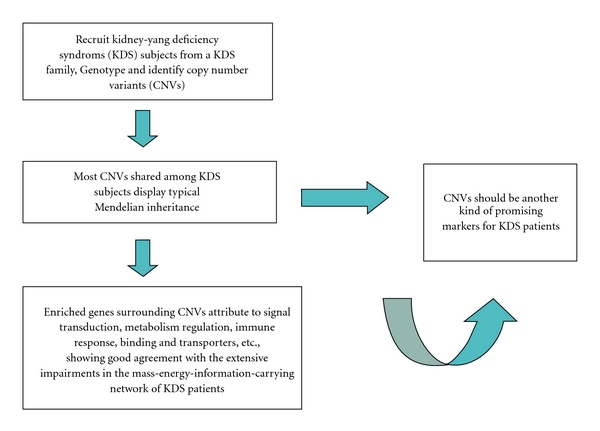
A hypothetical diagram demonstrating the relationships between KDS patients and CNVs.

In conclusion, this article reports the first set of CNVs involved in KDS, one of the principle TCM syndromes. These CNVs display a clear Mendelian inheritance. Genes located in CNV flanks are significantly enriched and generally consistent with key symptoms of KDS patients. These CNVs would greatly benefit the genetic and functional analysis of KDS. Hence, we propose a hypothetical diagram that demonstrates the relationships between KDS patients and their CNVs (Figure 3). Our data will contribute to a further understanding of the genetic basis of the KDS and will allow novel strategies for a rational therapy of this disease.

## Funding

National Natural Science Foundation of China (NSFC) (NO: 30371709 and NO: 30582288).

## Figures and Tables

**Table 1:** Statistic results of homologus and heterologous CNVs from a KDS family.

Copy number(s)	Number of homologous CNVs (%)	Number of heterologous CNVs (%)
CNV = 0	12 (100)	0 (0)
CNV = 1	433 (99.77)	1 (0.23)
CNV = 3	278 (76.58)	85 (23.42)
CNV = 4	20 (17.70)	93 (82.30)

**Table 2:** Genes mined within 100 bp of the CNVs of a KDS family.

CNV types	Functional class	Representative genes
Deleted CNVs	Binding and transporters	ZNF509, RNF150, DMRTC2, MYRIP, DDX26B, ZNF509, DMRTC2, ZNF509, MYRIP, FRMD4A, SCHIP1, DNAJC18, SDK1, CDH17, RP13-102H20.1, SLC5A9, MYRIP, ENTHD1
	Cell cycle	NEK7, PPP1R1C, MID1, FAM162A, RFC3, CTAGE5, RHOJ, CDC73, EIF4G3, CDKL1
	signal transduction	SDK1, LPHN3, FNDC3B, HAND2, MDGA1, MATR3, DST, MDGA1, CRADD, ARHGEF1, PKIB, NRG1, PIK3AP, ARHGAP25, GRAP2
	Immune response	C4orf7, CSN3, PROL1, GPX3, C1RL, GRAP2
Enlarged CNVs	Binding and transporters	ROR1, ERCC6, ALKBH8, C1orf83, KIAA0999, TUBA8, SLC28A2, LOC401898, KIAA2022, ZNF331, ZNF487, C1orf83, ELAC2, PCDH9, DNAJC3, LRRC50, ZNF208, LOC401898, LOC730087, ELAC2, CRB1, FSTL5, CDH19, TRPM3, TMTC1, SYT1, FSTL5, CPLX4, CDH19, DIAPH3, ERGIC2, LOC348751, COG2, SLC28A2
	Nervous development	ROR1, SEMA5A, SEMA5B, OPRM1, DYM, CNTN5, NTRK3, VIPR2, PCDH9
	Metabolism regulation	AHCYL1, PTTG2, PRIM2, HPGD, HPSE2, ALKBH8, PNLIPRP3, MBTPS1
	Biogenesis	SPATA5L1, ODF2, FMN1, SPTLC1, TBC1D1, HNT, MATN2, DGFC, GREM1, BMP
	Immune response	NTRK3, GPR177, ASTN2, CNTN5, GAB2, ELMOD1, DNAJC3, FER, HLA-DQB1

**Table 3:** Pathways derived from the genes located in the CNV flanks of a KDS family.

CNVs	Copy numbers	Related genes	Related pathways
rs2002594; rs1986617	1	FAM162A	Mitochondrial apoptotic cascades
rs10517547; rs10517548; rs2122642; rs2345041; rs1823535; rs1823536; rs1470724	1	LPHN3	G-protein-coupled-receptor proteolysis
rs764223	1	PGAM1	Glycolysis/gluconeogenesis; metabolism of carbohydrates
rs137964; rs10483201	1	GRAP2	T cell receptor signaling pathway
rs10491333	4	HLA-DQB1	Antigen processing and presentation
rs2895201	4	SPTLC1	Sphingolipid metabolism
rs1988981; rs1321555; rs1358815; rs1964875;	4	PRIM2	Purine and pyrimidine metabolism; telomere maintenance
rs3797819; rs10515399; rs1896582; rs1366081; rs9326759	4	FER	Adherens junction
rs342597; rs10507638; rs10507639	3	DIAPH3	Regulation of actin cytoskeleton
rs10520675; rs10520671; rs7167737; rs1948066; rs10520673; rs965313	3	NTRK3	MAPK pathway
rs6816712; rs2911940; rs10517654; rs10517655; rs7662187; rs10517658	3	PDGFC	Cytokine-cytokine receptor interaction; Focal adhesion; Gap junction
rs855858; rs855860; rs10489736	3	ROR1	transmembrane receptor protein tyrosine kinase signaling pathway
rs2912	3	GAB2	Fc epsilon RI signaling pathway
rs10491050; rs7092874; rs7904118	3	SORCS1	neuropeptide signaling pathway
rs10485060; rs9322451; rs1852629	3	OPRM1	Neuroactive ligand-receptor interaction
rs1060447; rs4919231; rs1932796; rs716838; rs2169462; rs10509722	3	HPSE2	Glycosaminoglycan degradation
